# Healthcare Access and Barriers Among Older Asian Americans: Cultural Influences and Systemic Challenges

**DOI:** 10.1093/geroni/igaf122.384

**Published:** 2025-12-31

**Authors:** Shinae Choi, Hee Lee, Hyunjin Noh, Peiyuan Zhang, Hannah Francis

**Affiliations:** The University of Alabama, Tuscaloosa, Alabama, United States; The University of Alabama, Tuscaloosa, Alabama, United States; The University of Alabama, Tuscaloosa, Alabama, United States; University of Maryland, Baltimore County, Baltimore, Maryland, United States; The University of Alabama, Tuscaloosa, Alabama, United States

## Abstract

Although nearly a quarter of the 19 million Asian immigrants in the U.S. call the South home, many still struggle to access and utilize healthcare services, revealing critical gaps in the system. This study investigated older Asian Americans’ access to healthcare services, utilization patterns, and quality of healthcare services received. We interviewed 15 Asian Americans, aged 50 or older, residing in West Alabama. Individual semi-structured interviews were conducted either virtually or in-person in English between July and November 2023. Each interview lasted 60-90 minutes. All interviews were recorded, transcribed, and thematically analyzed using open coding. Our analysis revealed six main themes on older Asian Americans’ healthcare services utilization: 1) dual cultural influences on healthcare service expectations, shaped by both the country of origin and the U.S., 2) hesitancy taking medication due to concerns about over-prescription, 3) long waiting and delayed care, and 4) navigating different healthcare logistics and high medical expenses; nearly all participants reported paying at check-out in their country of origin, where healthcare costs are known upfront. However, in the U.S., medical bills often arrive weeks or months later, making it difficult to anticipate and understand any out-of-pocket costs. Additional challenges addressed 5) hesitancy to ask questions during check-ups and 6) perceived discrimination from healthcare service providers. These challenges often lead to underutilization of services and unmet healthcare needs in the community. Our findings highlight critical needs for culturally tailored healthcare policies and targeted interventions to eliminate disparities, enhance accessibility, and improve health outcomes for older Asian Americans.

